# *N*-Benzyl-2-(2,4-dichloro­phen­oxy)acetamide

**DOI:** 10.1107/S1600536808029371

**Published:** 2008-09-20

**Authors:** Ming-Jun Chen, Yang-Wu Fu, Wen-Liang Dong, Zhu-Bo Li, Hua Zuo

**Affiliations:** a College of Chemical and Environmental Engineering, Chongqing Three Gorges University, Chongqing, 404000, People’s Republic of China; b Shandong University of Traditional Chinese Medicine, Jinan, 250355, People’s Republic of China; c College of Pharmaceutical Sciences, Southwest University, Chongqing, 400716, People’s Republic of China

## Abstract

In the title compound, C_15_H_13_Cl_2_NO_2_, the dihedral angle between the aromatic rings is 27.17 (11)°. In the crystal the molecules are linked by N—H⋯O hydrogen bonds.

## Related literature

For related literature, see: Li *et al.* (2008*a* ▶,*b* ▶).
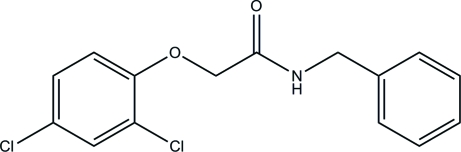


## Experimental

### 

#### Crystal data

C_15_H_13_Cl_2_NO_2_
*M* *_r_* = 310.16
Monoclinic, 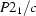

*a* = 4.7447 (6) Å
*b* = 26.821 (3) Å
*c* = 11.3962 (15) Å
β = 90.402 (2)°
*V* = 1450.2 (3) Å^3^
*Z* = 4
Mo *K*α radiation
μ = 0.45 mm^−1^
*T* = 298 (2) K
0.30 × 0.10 × 0.05 mm

#### Data collection

Bruker SMART APEXII CCD area-detector diffractometer
Absorption correction: multi-scan (*SADABS*; Bruker, 2005 ▶)*T*_min_ = 0.877, *T*_max_ = 0.978
8418 measured reflections
3254 independent reflections
2233 reflections with *I* > 2σ(*I*)
*R*_int_ = 0.027

#### Refinement

*R*[*F* ^2^ > 2σ(*F* ^2^)] = 0.039
*wR*(*F* ^2^) = 0.103
*S* = 1.03
3254 reflections
185 parameters
H atoms treated by a mixture of independent and constrained refinement
Δρ_max_ = 0.19 e Å^−3^
Δρ_min_ = −0.24 e Å^−3^

### 

Data collection: *APEX2* (Bruker, 2005 ▶); cell refinement: *SAINT* (Bruker, 2005 ▶); data reduction: *SAINT*; program(s) used to solve structure: *SIR97* (Altomare *et al.*, 1999 ▶); program(s) used to refine structure: *SHELXL97* (Sheldrick, 2008 ▶); molecular graphics: *SHELXTL* (Sheldrick, 2008 ▶); software used to prepare material for publication: *WinGX* (Farrugia, 1999 ▶).

## Supplementary Material

Crystal structure: contains datablocks I, New_Global_Publ_Block. DOI: 10.1107/S1600536808029371/cs2090sup1.cif

Structure factors: contains datablocks I. DOI: 10.1107/S1600536808029371/cs2090Isup2.hkl

Additional supplementary materials:  crystallographic information; 3D view; checkCIF report

## Figures and Tables

**Table 1:** Hydrogen-bond geometry (Å, °)

*D*—H⋯*A*	*D*—H	H⋯*A*	*D*⋯*A*	*D*—H⋯*A*
N—H0*A*⋯O2^i^	0.83 (2)	2.06 (2)	2.883 (2)	169 (2)

Symmetry code: (i) 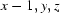.

**Fig. 1.**
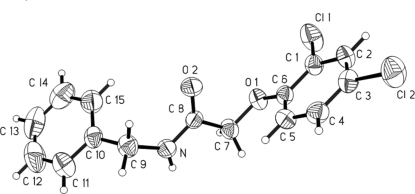
The molecular structure of the title compound showing displacement ellipsoids drawn at 50% probability level.

## supplementary crystallographic information

### Comment

The structure determination was performed as a part of a project on the interactions of small molecules with proteins. The single-crystal characterization should be valuable to understand such interactions. In our previous papers we reported single crystal structures of *N*-benzyl-2-(2-chloro-4-methylphenoxy)acetamide (Li *et al.*, 2008a) and *N*-benzyl-2-(2,6-dichlorophenoxy)acetamide (Li *et al.*, 2008b). In the title compound, C_15_H_13_Cl_2_NO_2_, all bond lengths and angles are normal. The dihedral angle between the two aryl rings is 27.17 (11)*^o^*. The molecules are connected via N-H···O hydrogen bonding into chains.

### Experimental

The solution of 2,4-dichlorophenol (1.0 mmol), *N*-benzyl-2-chloroacetamide (1.1 mmol), K_2_CO_3_ (1.1 mmol) and CH_3_CN (20 ml) was refluxed for 3 h. After completion of the reaction, the solution was cooled; solvent was evaporated under reduced pressure. The residue was poured into water and adjusted the pH to 6–7 with dilute hydrochloric acid (10%) and extracted with ethyl acetate, washed with brine and dried over anhydrous MgSO_4_ to obtain the corresponding crude product. The product was purified by column chromatography on silica gel using ethyl acetate as eluent (yield 87%). Crystals suitable for X-ray diffraction were obtained by slow evaporation of a solution of the solid dissolved in ethyl acetate/hexane at room temperatures for 4 days.

### Refinement

All H atoms were placed in geometrically calculated positions and refined using a riding model with C—H = 0.97 Å (for CH_2_ groups) and 0.96 Å (for CH_3_ groups), their isotropic displacement parameters were set to 1.2 times (1.5 times for CH_3_ groups) the equivalent displacement parameter of their parent atoms, except the N-H one which was freely refined.

### Crystal data

**Table:**

C_15_H_13_Cl_2_NO_2_	*F*(000) = 640
*M**_r_* = 310.16	*D*_x_ = 1.421 Mg m^−^^3^
Monoclinic, *P*2_1_/*c*	Mo *K*α radiation, λ = 0.71073 Å
Hall symbol: -P 2ybc	Cell parameters from 2023 reflections
*a* = 4.7447 (6) Å	θ = 2.4–24.3°
*b* = 26.821 (3) Å	µ = 0.45 mm^−^^1^
*c* = 11.3962 (15) Å	*T* = 298 K
β = 90.402 (2)°	Prism, colourless
*V* = 1450.2 (3) Å^3^	0.30 × 0.10 × 0.05 mm
*Z* = 4	

### Data collection

**Table:**

Bruker SMART CCD area-detector diffractometer	3254 independent reflections
Radiation source: fine-focus sealed tube	2233 reflections with *I* > 2σ(*I*)
graphite	*R*_int_ = 0.027
phi and ω scans	θ_max_ = 27.5°, θ_min_ = 1.5°
Absorption correction: multi-scan (PROGRAM? REFERENCE?)	*h* = −6→6
*T*_min_ = 0.878, *T*_max_ = 0.978	*k* = −26→34
8418 measured reflections	*l* = −14→14

### Refinement

**Table:**

Refinement on *F*^2^	Primary atom site location: structure-invariant direct methods
Least-squares matrix: full	Secondary atom site location: difference Fourier map
*R*[*F*^2^ > 2σ(*F*^2^)] = 0.039	Hydrogen site location: inferred from neighbouring sites
*wR*(*F*^2^) = 0.103	H atoms treated by a mixture of independent and constrained refinement
*S* = 1.03	*w* = 1/[σ^2^(*F*_o_^2^) + (0.0422*P*)^2^ + 0.2337*P*] where *P* = (*F*_o_^2^ + 2*F*_c_^2^)/3
3254 reflections	(Δ/σ)_max_ = 0.001
185 parameters	Δρ_max_ = 0.19 e Å^−^^3^
0 restraints	Δρ_min_ = −0.24 e Å^−^^3^

### Fractional atomic coordinates and isotropic or equivalent isotropic displacement parameters (Å^2^)

**Table:**

	*x*	*y*	*z*	*U*_iso_*/*U*_eq_	
N	−0.5859 (3)	0.13972 (6)	1.13239 (14)	0.0432 (4)	
H0A	−0.757 (4)	0.1456 (7)	1.1390 (16)	0.045 (6)*	
Cl1	−0.01187 (15)	0.32457 (2)	1.38966 (5)	0.0718 (2)	
Cl2	0.22803 (14)	0.39046 (2)	0.95534 (5)	0.0711 (2)	
O1	−0.3722 (3)	0.25520 (5)	1.27258 (11)	0.0467 (3)	
O2	−0.1562 (3)	0.17031 (5)	1.18000 (13)	0.0533 (4)	
C1	−0.0568 (4)	0.32072 (6)	1.23900 (15)	0.0419 (4)	
C2	0.0880 (4)	0.35291 (7)	1.16733 (16)	0.0470 (5)	
H2A	0.2087	0.3767	1.1991	0.056*	
C3	0.0511 (4)	0.34928 (7)	1.04765 (16)	0.0462 (5)	
C4	−0.1215 (4)	0.31367 (7)	0.99970 (17)	0.0496 (5)	
H4A	−0.1415	0.3112	0.9187	0.059*	
C5	−0.2659 (4)	0.28142 (7)	1.07243 (16)	0.0468 (5)	
H5A	−0.3834	0.2573	1.0398	0.056*	
C6	−0.2379 (4)	0.28461 (6)	1.19306 (15)	0.0386 (4)	
C7	−0.5631 (4)	0.21854 (7)	1.23010 (18)	0.0468 (5)	
H7A	−0.6842	0.2334	1.1707	0.056*	
H7B	−0.6816	0.2075	1.2941	0.056*	
C8	−0.4137 (4)	0.17379 (6)	1.17797 (15)	0.0370 (4)	
C9	−0.4894 (4)	0.09348 (7)	1.08006 (17)	0.0507 (5)	
H9A	−0.2910	0.0966	1.0615	0.061*	
H9B	−0.5912	0.0880	1.0071	0.061*	
C10	−0.5293 (4)	0.04881 (7)	1.15846 (17)	0.0459 (5)	
C11	−0.7149 (5)	0.01174 (9)	1.1278 (2)	0.0726 (7)	
H11A	−0.8146	0.0140	1.0574	0.087*	
C12	−0.7554 (7)	−0.02898 (10)	1.2005 (3)	0.0908 (9)	
H12A	−0.8836	−0.0536	1.1788	0.109*	
C13	−0.6107 (7)	−0.03343 (10)	1.3027 (3)	0.0872 (9)	
H13A	−0.6395	−0.0608	1.3514	0.105*	
C14	−0.4209 (6)	0.00298 (11)	1.3337 (2)	0.0822 (8)	
H14A	−0.3190	0.0001	1.4034	0.099*	
C15	−0.3801 (5)	0.04402 (9)	1.2619 (2)	0.0640 (6)	
H15A	−0.2512	0.0685	1.2838	0.077*	

### Atomic displacement parameters (Å^2^)

**Table:**

	*U*^11^	*U*^22^	*U*^33^	*U*^12^	*U*^13^	*U*^23^
N	0.0327 (9)	0.0426 (9)	0.0543 (10)	0.0032 (7)	0.0025 (7)	−0.0059 (7)
Cl1	0.1088 (5)	0.0673 (4)	0.0393 (3)	−0.0261 (3)	−0.0006 (3)	−0.0106 (2)
Cl2	0.0869 (5)	0.0663 (4)	0.0604 (3)	−0.0168 (3)	0.0117 (3)	0.0131 (3)
O1	0.0517 (8)	0.0405 (7)	0.0479 (7)	−0.0087 (6)	0.0045 (6)	−0.0019 (6)
O2	0.0307 (7)	0.0527 (8)	0.0764 (10)	0.0030 (6)	0.0010 (6)	−0.0051 (7)
C1	0.0511 (11)	0.0370 (10)	0.0376 (9)	−0.0001 (8)	0.0002 (8)	−0.0071 (8)
C2	0.0539 (12)	0.0377 (10)	0.0492 (11)	−0.0072 (9)	0.0011 (9)	−0.0065 (8)
C3	0.0506 (11)	0.0407 (10)	0.0475 (11)	0.0004 (9)	0.0053 (9)	0.0029 (9)
C4	0.0584 (13)	0.0515 (12)	0.0388 (10)	0.0030 (10)	−0.0048 (9)	0.0002 (9)
C5	0.0488 (11)	0.0439 (11)	0.0478 (11)	−0.0042 (9)	−0.0083 (9)	−0.0046 (9)
C6	0.0394 (10)	0.0320 (9)	0.0445 (10)	0.0029 (8)	0.0014 (8)	−0.0020 (8)
C7	0.0377 (10)	0.0403 (10)	0.0626 (12)	−0.0041 (8)	0.0075 (9)	−0.0021 (9)
C8	0.0329 (10)	0.0354 (9)	0.0428 (10)	0.0010 (7)	0.0025 (7)	0.0057 (7)
C9	0.0542 (13)	0.0478 (12)	0.0501 (11)	0.0018 (9)	0.0049 (9)	−0.0093 (9)
C10	0.0456 (11)	0.0404 (10)	0.0519 (11)	0.0043 (9)	0.0101 (9)	−0.0087 (9)
C11	0.0747 (17)	0.0613 (15)	0.0816 (17)	−0.0152 (13)	−0.0013 (13)	−0.0055 (13)
C12	0.098 (2)	0.0560 (16)	0.119 (3)	−0.0247 (15)	0.020 (2)	−0.0025 (16)
C13	0.103 (2)	0.0540 (16)	0.105 (2)	0.0148 (16)	0.0368 (19)	0.0151 (15)
C14	0.096 (2)	0.0809 (19)	0.0693 (17)	0.0239 (17)	0.0031 (14)	0.0134 (15)
C15	0.0697 (15)	0.0590 (14)	0.0632 (14)	0.0012 (11)	−0.0021 (12)	−0.0024 (11)

### Geometric parameters (Å, °)

**Table:**

N—C8	1.329 (2)	C7—C8	1.517 (2)
N—C9	1.451 (2)	C7—H7A	0.9700
N—H0A	0.83 (2)	C7—H7B	0.9700
Cl1—C1	1.7318 (18)	C9—C10	1.507 (3)
Cl2—C3	1.7448 (19)	C9—H9A	0.9700
O1—C6	1.363 (2)	C9—H9B	0.9700
O1—C7	1.420 (2)	C10—C11	1.372 (3)
O2—C8	1.225 (2)	C10—C15	1.377 (3)
C1—C2	1.376 (3)	C11—C12	1.385 (4)
C1—C6	1.394 (2)	C11—H11A	0.9300
C2—C3	1.377 (3)	C12—C13	1.353 (4)
C2—H2A	0.9300	C12—H12A	0.9300
C3—C4	1.369 (3)	C13—C14	1.373 (4)
C4—C5	1.383 (3)	C13—H13A	0.9300
C4—H4A	0.9300	C14—C15	1.386 (3)
C5—C6	1.383 (2)	C14—H14A	0.9300
C5—H5A	0.9300	C15—H15A	0.9300
			
C8—N—C9	123.59 (16)	O2—C8—N	124.41 (16)
C8—N—H0A	115.6 (14)	O2—C8—C7	121.43 (16)
C9—N—H0A	120.7 (14)	N—C8—C7	114.16 (15)
C6—O1—C7	118.32 (14)	N—C9—C10	113.22 (16)
C2—C1—C6	121.47 (17)	N—C9—H9A	108.9
C2—C1—Cl1	119.52 (14)	C10—C9—H9A	108.9
C6—C1—Cl1	119.00 (14)	N—C9—H9B	108.9
C1—C2—C3	118.88 (17)	C10—C9—H9B	108.9
C1—C2—H2A	120.6	H9A—C9—H9B	107.7
C3—C2—H2A	120.6	C11—C10—C15	118.4 (2)
C4—C3—C2	121.07 (18)	C11—C10—C9	120.5 (2)
C4—C3—Cl2	119.33 (15)	C15—C10—C9	121.05 (19)
C2—C3—Cl2	119.60 (15)	C10—C11—C12	120.7 (3)
C3—C4—C5	119.63 (18)	C10—C11—H11A	119.7
C3—C4—H4A	120.2	C12—C11—H11A	119.7
C5—C4—H4A	120.2	C13—C12—C11	120.9 (3)
C6—C5—C4	120.84 (18)	C13—C12—H12A	119.6
C6—C5—H5A	119.6	C11—C12—H12A	119.6
C4—C5—H5A	119.6	C12—C13—C14	119.1 (3)
O1—C6—C5	125.67 (16)	C12—C13—H13A	120.5
O1—C6—C1	116.25 (15)	C14—C13—H13A	120.5
C5—C6—C1	118.09 (17)	C13—C14—C15	120.5 (3)
O1—C7—C8	112.50 (14)	C13—C14—H14A	119.8
O1—C7—H7A	109.1	C15—C14—H14A	119.8
C8—C7—H7A	109.1	C10—C15—C14	120.4 (2)
O1—C7—H7B	109.1	C10—C15—H15A	119.8
C8—C7—H7B	109.1	C14—C15—H15A	119.8
H7A—C7—H7B	107.8		
			
C6—C1—C2—C3	0.3 (3)	C9—N—C8—O2	0.7 (3)
Cl1—C1—C2—C3	179.74 (15)	C9—N—C8—C7	−178.70 (16)
C1—C2—C3—C4	−1.4 (3)	O1—C7—C8—O2	3.5 (3)
C1—C2—C3—Cl2	178.90 (14)	O1—C7—C8—N	−177.05 (15)
C2—C3—C4—C5	1.3 (3)	C8—N—C9—C10	103.3 (2)
Cl2—C3—C4—C5	−178.97 (15)	N—C9—C10—C11	113.8 (2)
C3—C4—C5—C6	−0.1 (3)	N—C9—C10—C15	−66.5 (3)
C7—O1—C6—C5	−1.4 (2)	C15—C10—C11—C12	1.4 (3)
C7—O1—C6—C1	178.78 (15)	C9—C10—C11—C12	−179.0 (2)
C4—C5—C6—O1	179.23 (17)	C10—C11—C12—C13	−0.8 (4)
C4—C5—C6—C1	−1.0 (3)	C11—C12—C13—C14	−0.3 (4)
C2—C1—C6—O1	−179.29 (16)	C12—C13—C14—C15	0.7 (4)
Cl1—C1—C6—O1	1.3 (2)	C11—C10—C15—C14	−0.9 (3)
C2—C1—C6—C5	0.9 (3)	C9—C10—C15—C14	179.4 (2)
Cl1—C1—C6—C5	−178.57 (14)	C13—C14—C15—C10	−0.1 (4)
C6—O1—C7—C8	75.3 (2)		

### Hydrogen-bond geometry (Å, °)

**Table:**

*D*—H···*A*	*D*—H	H···*A*	*D*···*A*	*D*—H···*A*
N—H0A···O2^i^	0.83 (2)	2.06 (2)	2.883 (2)	169 (2)

Symmetry codes: (i) *x*−1, *y*, *z*.
